# Stable and Effective Online Monitoring and Feedback Control of PCDD/F during Municipal Waste Incineration

**DOI:** 10.3390/molecules26144290

**Published:** 2021-07-15

**Authors:** Shijian Xiong, Fanjie Shang, Ken Chen, Shengyong Lu, Shaofu Tang, Xiaodong Li, Kefa Cen

**Affiliations:** 1 State Key Laboratory of Clean Energy Utilization, Zhejiang University, Hangzhou 310027, China; 11827072@zju.edu.cn (S.X.); 21927009@zju.edu.cn (K.C.); lixd@zju.edu.cn (X.L.); kfcen@zju.edu.cn (K.C.); 2 Zhejiang Fuchunjiang Environmental Technology Research Co., Ltd., Hangzhou 311401, China; shangfanjie@163.com (F.S.); shaofutang263@163.com (S.T.)

**Keywords:** online monitoring, diagnosis, PCDD/F, incineration, feedback control

## Abstract

For the long-term operation of municipal solid waste incineration (MSWI), online monitoring and feedback control of polychlorinated dibenzo-*p*-dioxin and dibenzofuran (PCDD/F) can be used to control the emissions to national or regional standards. In this study, 500 PCDD/F samples were determined by thermal desorption gas chromatography coupled to tunable-laser ionization time-of-flight mass spectrometry (TD-GC-TLI-TOFMS) for 168 h. PCDD/F emissions range from 0.01 ng I-TEQ/Nm^3^ to 2.37 ng I-TEQ/Nm^3^, with 44% of values below 0.1 ng I-TEQ/Nm^3^ (the national standard). In addition, the temperature of the furnace outlet, bed pressure, and oxygen content are considered as key operating parameters among the 13 operating parameters comprising four temperature parameters, four pressure parameters, four flow parameters, and oxygen content. More specifically, maintaining the furnace outlet temperature to be higher than 800 °C, or bed pressure higher than 13 kPa, or the oxygen content stably and above 10% are effective methods for reducing PCDD/F emissions. According to the analysis of the Pearson coefficients and maximal information coefficients, there is no significant correlation between operating parameters and PCDD/F I-TEQ. Only when there is a significant change in one of these factors will the PCDD/F emissions also change accordingly. The feedback control of PCDD/F emissions is realized by adjusting the furnace outlet temperature, bed temperature, and bed pressure to control the PCDD/F to be less than 0.1 ng I-TEQ/Nm^3^.

## 1. Introduction

Nowadays, incineration is considered to be the preferred technology to dispose of municipal solid waste (MSW) in China [1]. In the future, all MSW in in Zhejiang province, China, will be disposed of by incineration. However, the toxic pollutants emitted from MSW incineration, especially polychlorinated dibenzo-p-dioxins and dibenzofurans (PCDD/F), are risky to the environment and human health. Furthermore, the measurement of PCDD/F involves sampling, extraction, purification, and analysis with high-resolution gas chromatography/high-resolution mass spectrometry (HRGC/HRMS) [2,3]. Hence, the traditional analysis procedure for PCDD/F from industrial incineration takes at least a week. Additionally, the cost of the traditional measurement method is high, resulting in a measurement cycle of once a year. Nonetheless, the long-term sampling of PCDD/F has been verified as an effective monitoring method for the total PCDD/F emission over a month [4]. Though the standard method and long-term sampling ensure the accuracy of measurements, the time lag hardly meets the public concern and prevents the development of rapid feedback control [5].

Due to the diversity of the 210 PCDD/F congeners and the parts per quadrillion concentrations, online monitoring of PCDD/F concentrations can be realized by measuring the indicators with good correlation and higher concentrations. Based on a previous study [6], the indicators included chlorophenols, chlorobenzene [7], and polycyclic aromatic hydrocarbons (PAH) [8]. In addition, the above indicators have been successfully determined by resonance-enhanced multi-photo ionization coupled to time-of-flight mass spectrometry (REMPI-TOFMS) [9,10], with a good correlation (R-square > 0.7) with PCDD/F emissions. Considering the difficulty of ionization and the correlation with PCDD/F emissions, 1,2,4-trichlorobenzene (1,2,4-TrCBz) is selected as the best indicator with a high correlation coefficient (R-square > 0.9) [11,12] with the international toxic equivalent quantity (I-TEQ). Hence, the online monitoring of PCDD/F emissions was realized by measuring the 1,2,4-TrCBz concentration in flue gas during MSW incineration, through thermal desorption gas chromatography (TD-GC)-REMPI-TOFMS [5,13]. Moreover, an accurate correlation model between 1,2,4-TrCBz and I-TEQ was constructed regardless of the change of waste composition and operating parameters [14]. Though the specific PCDD/F congeners were detected by vacuum ultraviolet (VUV) single-photon ionization (SPI) ion trap (IT) (VUV-SPI-IT)-TOFMS [15] or resonance ionization with multi-mirror photo accumulation (RIMMPA)-TOFMS [16], the measurement cycle was 2–6 h, resulting in limited feedback responsiveness. Therefore, the method using 1,2,4-TrCBz as an indicator is considered to be the most promising way to realize online monitoring of PCDD/F emissions from industrial incineration.

The stable and continuous online monitoring of PCDD/F is crucial for the MSW incinerator rather than the experiments for 2–3 days in previous studies [5,13]. Due to the fluctuation of operation parameters during MSW incineration (MSWI), PCDD/F emissions can increase or decrease at short notice. As for the long-term operation of the MSW incinerator, there is no study reporting a change of PCDD/F emissions in a short time (5–10 min) rather than the average value of 2–4 h. An accurate correlation between operating parameters and PCDD/F emissions is key to the diagnosis and feedback control of PCDD/F but difficult to be constructed, due to being limited by the defects of the standard method. As for the industrial managers and government, the optimal operating parameters for PCDD/F emissions are crucial for controlling the emissions to national or regional standards (0.1 ng I-TEQ/Nm^3^ or 0.05 ng I-TEQ/Nm^3^). Furthermore, numerous studies constructed the control strategy to reduce PCDD/F emissions by adding inhibitors [17]/active carbon [18], cleaning the accumulated fly ash [19], and reducing the chlorine content of waste [20]. However, the effect of temperature and oxygen content on the formation of PCDD/F was only investigated on a reactor in the laboratory [21,22] or pilot scale sintering plant [23] rather than a full-scale incinerator. For the full-scale MSW incinerator, the cost for sampling PCDD/F is high, and adjusting the operating parameters is difficult; it is both difficult and meaningful to investigate the correlation between operating parameters and PCDD/F emissions.

In the present study, continuous online PCDD/F measurement was carried out at the stack of a full-scale MSW incinerator, lasting 168 h. Firstly, calibration and validation of the equipment were constructed to ensure accuracy and effectiveness. Then, a total of 500 stack gas samples were collected and determined by TD-GC-REMPI-TOFMS. The effect of operating parameters on the PCDD/F formation was investigated, which include temperature, pressure, oxygen content, and mass transfer rate. Moreover, statistical methods were applied to analyze the correlation in depth. Finally, the diagnosis and feedback control of PCDD/F were realized in the MSW incinerator with the same air pollution control devices (APCD). The study is conducive to the development and application of the online monitoring of PCDD/F. In addition, PCDD/F emissions can be controlled to the national standard in time by rapid feedback control.

## 2. Materials and Methods

### 2.1. Experimental Procedure

**Figure 1:**
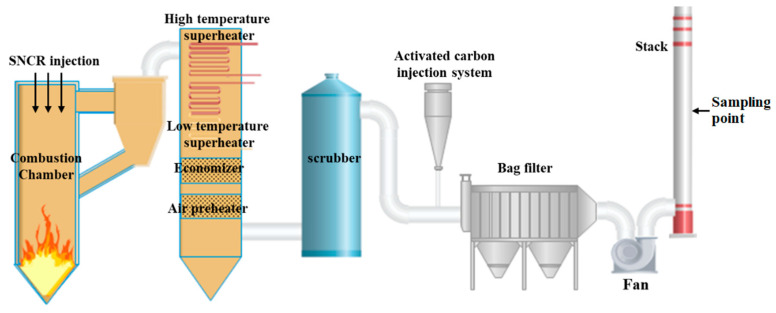
Schematic diagram of the MSW incinerator: sampling point at the stack.

First, the continuous and online measurement of PCDD/F was carried out at the stack of a municipal solid waste incinerator in Zhejiang Province. The capacity of the circulating fluidized bed (CFB) incinerator was 400 tons/day, and the mixing ratio of coal to raw refuse was 2–8. The air pollution control devices (APCD) included selective non-catalytic reduction (SNCR), semi-dry desulfurization (SDD), active carbon injection (ACI), and bag filter (BF). The schematic of the CFB and the sampling details are described in Figure 1. In order to ensure the continuous and stable operation of TD-GC-TLI-TOFMS, a special room was built to maintain constant temperature and humidity (temperature of 23 °C, relative humidity of 40%) and a certain degree of cleanliness (100,000 level) so as to avoid the impact of the external environment. Then, the diagnosis and feedback control experiments were carried out in another CFB, with 1200 tons/day capacity, which was fed by the refuse-derived fuel (RDF). The APCD of the newly built CFB was the same as the previous incinerator.

As for the stable and continuous monitoring of PCDD/F emissions, the international toxicity equivalent (I-TEQ) was selected to describe PCDD/F emissions at the stack. The experiments started at 9:00 a.m. on 19 March and ended at 9:00 a.m. on 26 March, for a total of 168 h. During this period, the TD-GC-TLI-TOFMS system performed a backflush cleaning procedure for approximately 1 h every 8 h, and ran continuously the rest of the time. The sampling time for each flue gas sample was 15 min, and the sampling flow rate was 10 mL/min. As for the diagnosis and feedback control of PCDD/F, the sampling time was 15 min. The operating parameters of the incinerator were directly collected from the Distributed Control System (DCS) and Continuous Emission Monitoring System (CEMS). The operating parameters included the temperature, pressure, flow, oxygen content, and feeding conditions.

### 2.2. Analysis Method

The flue gas was collected into the purification module at a flow rate of 3–10 L/min by the sampling pump. After the heating filter and Nafion drying tube, most of the dust particles and moisture were removed. Then, it flowed to the multipored valve group, discharging to the atmosphere. In this period, the Nafion tube was heated to about 100 °C, and all other sampling pipelines and heating filters were heated to about 200 °C to avoid condensation of 1,2,4-TrCBz or adsorption on the tube wall.

An accurate 1,2,4-TrCBz concentration was detected by the homemade instrument named tunable-laser ionization (TLI)-TOFMS, based REMPI. The instrument consists of a pulsed Nd: YAG optical parametric oscillator (OPO) laser with frequency doubling module (RADIANT, OPOTEK) and time-of-flight mass spectrometer (CTF10, STEFAN KAESDORF). The primary component of TD-GC-TLI-TOFMS is illustrated in Figure S1 (Supplementary Materials). First, the stack gas was sampled via a heated 1/4″ stainless steel tube (T = 180 °C) by a sampling pump. Second, after particles were removed by the sampling probe, a fraction (50 mL/min) was extracted through a heated 1/4″ stainless steel tube (T = 180 °C) from the mainstream. Third, the stack gas was absorbed by the TD device, with the operating condition that the absorbing and desorbing temperature of the trap was 30–300 °C. Fourth, the desorbed gas went to GC (Trace1300, Thermo Fisher) through a stainless capillary tube at 200 °C. Fifth, the gas separated after GC was extracted into the TOFMS through a pulsed microfluidic valve, with a supersonic molecular beam. Sixth, the ionization and detection of 1,2,4-TrCBz were carried out by a (1 + 1′) TLI process (284 and 213 nm). Then, the ions were separated for the purpose of the mass-to-charge ratio during the process to the microchannel plate (MCP) detector; the digitized MCP signals and their corresponding times were acquired by the data acquisition. Finally, the detection signal was transferred to a computer, and then the concentration values of the 1,2,4-TrCBz and the corresponding PCDD/F I-TEQ were calculated through processing and analysis with LabVIEW 2017. PCDD/F I-TEQ is calculated by the equation “y = 0.2752x + 0.0398”, where y represents the PCDD/F I-TEQ and x represents the concentration of 1,2,4-TrCBz.

### 2.3. Quality Control and Quality Assurance

The TD-GC-TLI-TOFMS system was calibrated daily by the 1,2,4-TrCBz calibration standard, with the concentration of 1,2,4-TrCBz ranging from 2.5 parts per billion by volume (ppbv) to 10 ppbv. The Relative Standard Deviation (RSD) of thermal desorption introduction was less than 2%, and the RSD of repeat measurements of the system was less than 5%. Moreover, the detection limit of 1,2,4-TrCBz in the stack gas was 4 parts per trillion by volume, on the condition that the sample volume was 1000 mL at a signal-to-noise ratio (S/N) of 3 [13,24]. To ensure the accuracy of the measurements, TD-GC-TLI-TOFMS was calibrated before, during, and after the experiment.

**Figure 2:**
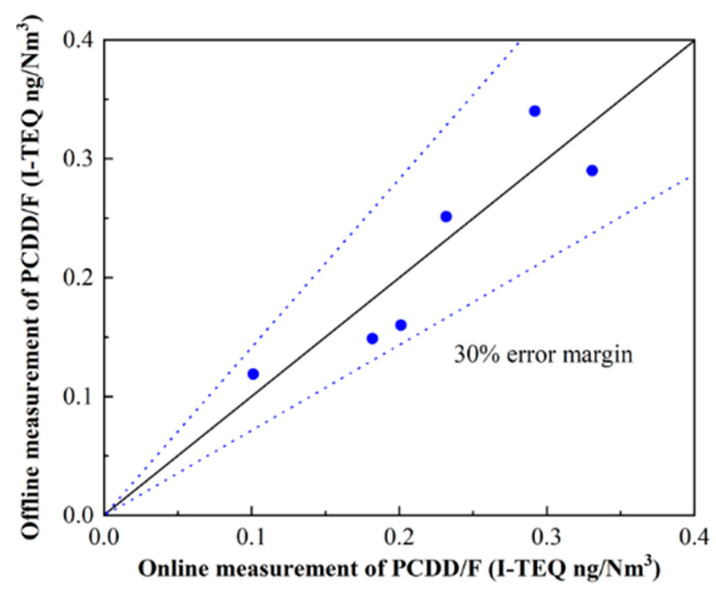
Comparison between online measurement and offline measurement of PCDD/F.

Furthermore, the validation between the prediction and the standard measurement was carried out before the continuous measurement. The standard measurement for PCDD/F of the flue gas was constructed by the EPA 23a, considered to be “offline measurement” due to a long lag time (more than 1 week). For the measurement of PCDD/F by the traditional method (EPA 23), the flue gas was collected utilizing an isokinetic sampler (Model KNJ23, KNJ, Korea). The sampling, pretreatment, and analysis followed the EPA method 1613. The details of the analysis method have been described in previous studies [3,25]. The relative error between online measurement and offline measurement was kept within 30% (Figure 2). Therefore, the accuracy of online detection and prediction of PCDD/F was reliable.

### 2.4. Statistical Analysis

The Pearson coefficient is universally used to measure linear correlation using the following equation:

(1) $$r=\frac{\sum_{i=1}^{n}\left(X_{i}-\bar{X}\right)\left(Y_{i}-\bar{Y}\right)\;}{\sqrt{\sum_{i=1}^{n}{\left(X_{i}-\bar{X}\right)}^{2}}\sqrt{\sum_{i=1}^{n}{\left(Y_{i}-\bar{Y}\right)}^{2}}}$$

The maximal information coefficient [26] is used to measure the dependence between two discrete variables whether linear or not, with fairness and symmetry.

(2) $$I\left(x,y\right)=\int^{\;}p\left(x,y\right)\log_{2}\frac{p\left(x,y\right)}{p\left(x\right)p\left(y\right)}dxdy\approx \underset{X,Y}{\sum }p\left(X,Y\right)\log_{2}\frac{p\left(X,Y\right)}{p\left(X\right)p\left(Y\right)}s$$

(3) $$MIC\left(X,Y\right)=\underset{\left|X\right|\left|Y\right|<B}{\max}\frac{I\left(X,Y\right)}{\log_{2}\left(\min\left(\left|X\right|,\left|Y\right|\right)\right)}$$

where *I*(*X*,*Y*) is the mutual information for two random variables *X* and *Y*. *B* is a parameter to limit the interval grid number, approximately 0.6 times the amount of data. The Pearson coefficient and maximal information coefficient between PCDD/F emissions and operating parameters were calculated in MATLAB 2020b. Furthermore, to investigate the relationship between operating parameters and PCDD/F I-TEQ, principal component analysis (PCA) was adopted.

## 3. Results and Discussion

### 3.1. PCDD/F Concentration

**Figure 3:**
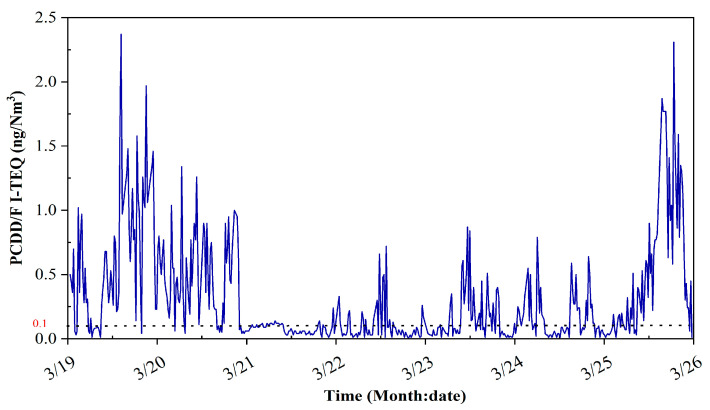
PCDD/F I-TEQ during the 168 h experiment.

Figure 3 shows all the results for PCDD/F I-TEQ during the continuous 168 h experiment. A total of 500 samples of stack gas were collected and measured throughout the procedure of the experiment. The average value of the calculated PCDD/F I-TEQ was 0.272 ng I-TEQ/Nm^3^. Among them, the calculated PCDD/F I-TEQ of 226 samples was less than 0.1 ng I-TEQ/Nm^3^ (the legislation limit of the national standard), accounting for approximately 44%. A drastic fluctuation of PCDD/F emissions could be observed. The results indicate that the concern regarding PCDD/F emissions needs to be transferred to long-term online monitoring rather than the measurement for a certain period.

The feedstock suspension happened frequently, and the intermission varied from minutes to hours. Remarkably, the longest suspension occurred from 07:30 a.m. on 21 March to 0:05 a.m. on 22 March, resulting in drastically changing operating parameters. Operating conditions of the incinerator such as the temperature of the furnace outlet, the oxygen content of the furnace outlet, and bed temperature were recorded every 10 min. The statistics of operating parameters is shown in Table 1. The average temperature of the furnace outlet was 835.6 °C, ranging from 686.9 °C to 967.1 °C. The average bed temperature was 780.7 °C, ranging from 261.1 °C to 908.4 °C. The trend of the furnace outlet temperature is similar to bed temperature. The average oxygen content of the furnace outlet was 8.82%, ranging from 0 to 20.02%. The results indicate the fluctuating operating conditions and the unstable combustion during the experiment. High levels of PCDD/F can be formed during experiments due to the failure of continuous and stable waste feeding.

**Figure 4:**
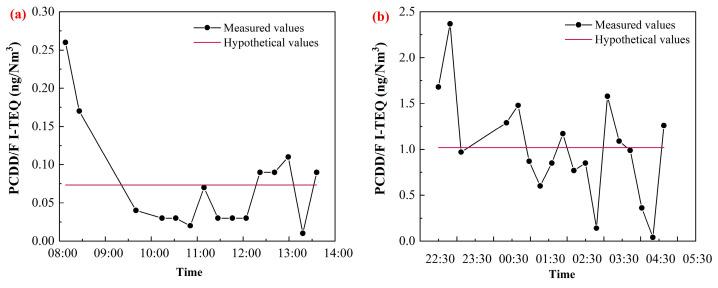
Comparison of hypothetical values of offline measurement and online measurement; hypothetical values represent the average values in continuous 6 h by TD-GC-REMPI-TOFMS. (**a**) low emissions under stable operation (**b**) high emissions under fluctuant operation.

Figure 4 shows the comparison between the hypothetical results to simulate offline measurement and the results by online measurement. The hypothetical values were obtained by calculating the average within 6 consecutive hours, which were measured online by TD-GC-TLI-TOFMS. The reason for this is that, in general, three hypothetical parallel samples were collected continuously within 6 h in the offline measurement. For example, the hypothetical value in Figure 4 is the average of the 15 values measured by the online method. Deduced from Figure 4, offline measurement cannot describe the change of PCDD/F emissions with the change of operating parameters. The hypothetical value for the high level of PCDD/F is 1.02 ng I-TEQ/Nm^3^. On the other hand, the hypothetical value for the low level of PCDD/F is only 0.07 ng I-TEQ/Nm^3^. It is obvious that the emissions measured by the offline method can only represent the average of PCDD/F emissions during the sampling period. They cannot reflect the dramatic changes of PCDD/F emissions from MSWI. Furthermore, the online measurement of PCDD/F provides the PCDD/F emission value in 15 min, which truly reflects the change of dioxin emissions over time. Therefore, it is the basis for the realization of feedback control for PCDD/F emissions.

### 3.2. Effect of Operating Parameters

Oxygen content, temperature, and pressure have been reported as the key parameters that influence PCDD/F formation [27]. A previous study reported the effect of oxygen on the formation of PCDD/F, including three aspects [28]: (1) promoting the formation of C-Cl bonds; (2) promoting the carbon-oxygen complexes by reducing the activation energy; (3) providing highly active chlorine in the Deacon reaction. In addition, the higher oxygen level was conducive to PCDD/F formation in laboratory-scale and full-scale incinerators [29,30]. As for temperature and pressure, the reaction rate and activation energy are decided by the two parameters if the reaction is fixed based on the Arrhenius equation. The effects of the above parameters on the formation of PCDD/F for the full-scale incinerator are discussed below.

#### 3.2.1. Effect of Temperature

**Figure 5:**
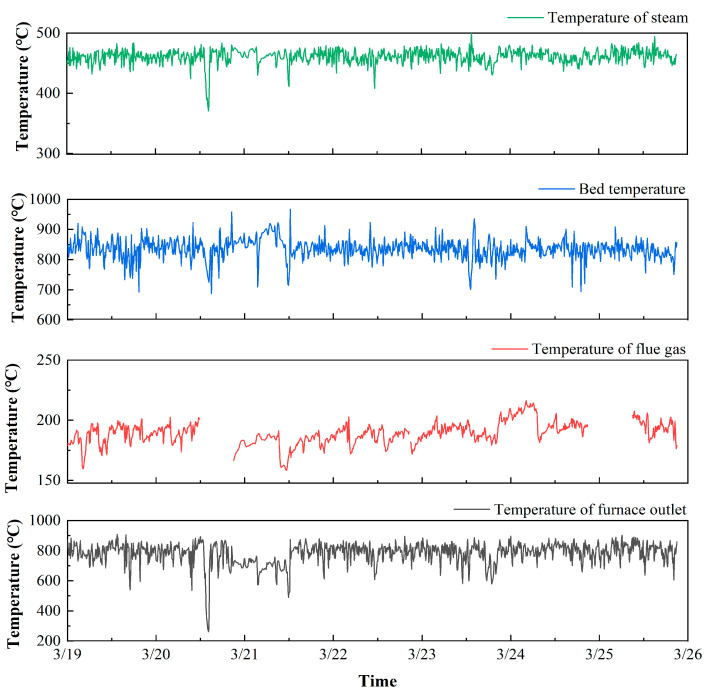
The trend for the temperatures of furnace outlet, bed, steam, and flue gas.

**Figure 6:**
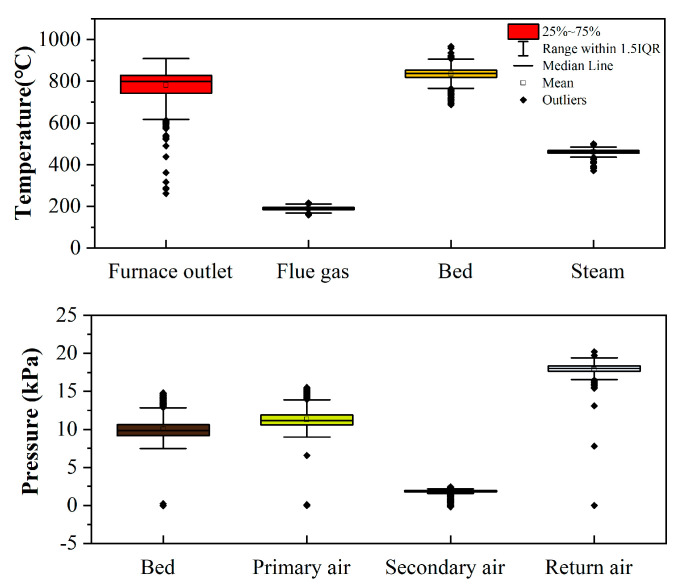
Comparison of the statistics for the temperature of furnace outlet, flue gas, bed, and steam, the pressure of bed, primary air, secondary air, and return air.

As shown in Figure 5, the fluctuating ranges of the furnace outlet temperature (647 °C) and bed temperature (280 °C) are larger than those of the flue gas temperature (58 °C) and the steam temperature (130 °C). The comparison of the statistics for the temperature of the furnace outlet, flue gas, bed, and steam is illustrated in Figure 6. The distributions for the temperature of the flue gas, bed, and steam are Gaussian, excluding the temperature of the furnace outlet. All outliers of the furnace outlet temperatures are less than 75% of the mean. The result indicates that part of the minimum values of the furnace outlet temperatures below 75% of the mean are abnormal conditions, resulting in high levels of pollutants. Corresponding to PCDD/F emissions, the trends of furnace outlet temperature and bed temperature are opposite to the trend of PCDD/F emissions on 20 March. As for flue gas temperature and steam temperature, there is almost no correspondence with PCDD/F emissions. These results indicate that furnace outlet temperature and bed temperature can be considered to be the key operating parameters controlling PCDD/F emissions.

For specific daily analysis (Table S1, Supplementary Materials), it can be deduced that the PCDD/F emissions decrease under the condition that the temperature of furnace outlet is maintained to be below 800 °C during the following times: 19 March (10:50–11:40, 12:10–12:20, 20:00–20:06); 20 March (22:30–22:50); 22 March (9:50–12:00, 13:30–15:30, 16:30–16:50); 23 March (22:00–22:30); 25 March (9:00–11:00, 13:10–13:50). On the other hand, PCDD/F emissions increase under the condition that the temperature of the furnace outlet is less than 700 °C. Therefore, maintaining the temperature of the furnace outlet to be higher than 800 °C is an effective method to reduce PCDD/F emissions.

#### 3.2.2. Effect of Pressure

**Figure 7:**
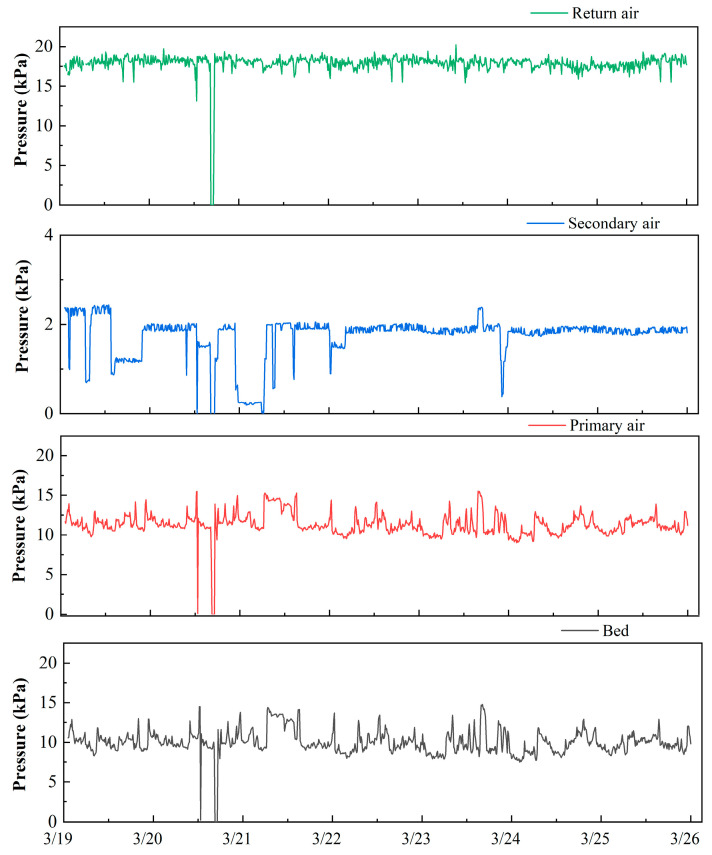
The trend for the pressure of the bed, primary air, secondary air, and return air.

As shown in Figure 7, the trend of bed pressure is consistent with that of primary air pressure. The pressure of the secondary air was kept stably with a mean of 2 kPa. The pressure of the return air was generally kept stably with a mean of 18 kPa, excluding two outliers (0.5 kPa). These results indicate that bed pressure is determined by the primary air pressure. The comparison of the statistics for the pressure of the bed, primary air, secondary air, and return air is illustrated in Figure 6. The distribution of bed pressure is the same as primary air pressure, with vast outliers larger than 125% of the mean. The distributions of the secondary air pressure and the return air pressure are both the left distribution. Theoretically, bed pressure can reflect the flow rate of air through the waste in the furnace. Hence, it is better to consider bed pressure to be the key operating parameter rather than primary air pressure.

Apart from the analysis of key operating parameters, the effect of bed pressure on the PCDD/F emissions is discussed below. Corresponding to the PCDD/F emissions on 21 March, the PCDD/F emissions were less than 0.1 ng I-TEQ/Nm^3^ when the bed pressure was larger than 13 kPa. This result indicates that a high bed pressure with a high mass transfer rate between air and waste can reduce PCDD/F emissions. However, the PCDD/F emissions were larger than 1 ng I-TEQ/Nm^3^ under the condition that the bed pressure was occasionally above the mean. This result indicates that the bed pressure is a more important parameter than the decisive parameter.

#### 3.2.3. Effect of Oxygen Content and Mass Transfer Rate

**Figure 8:**
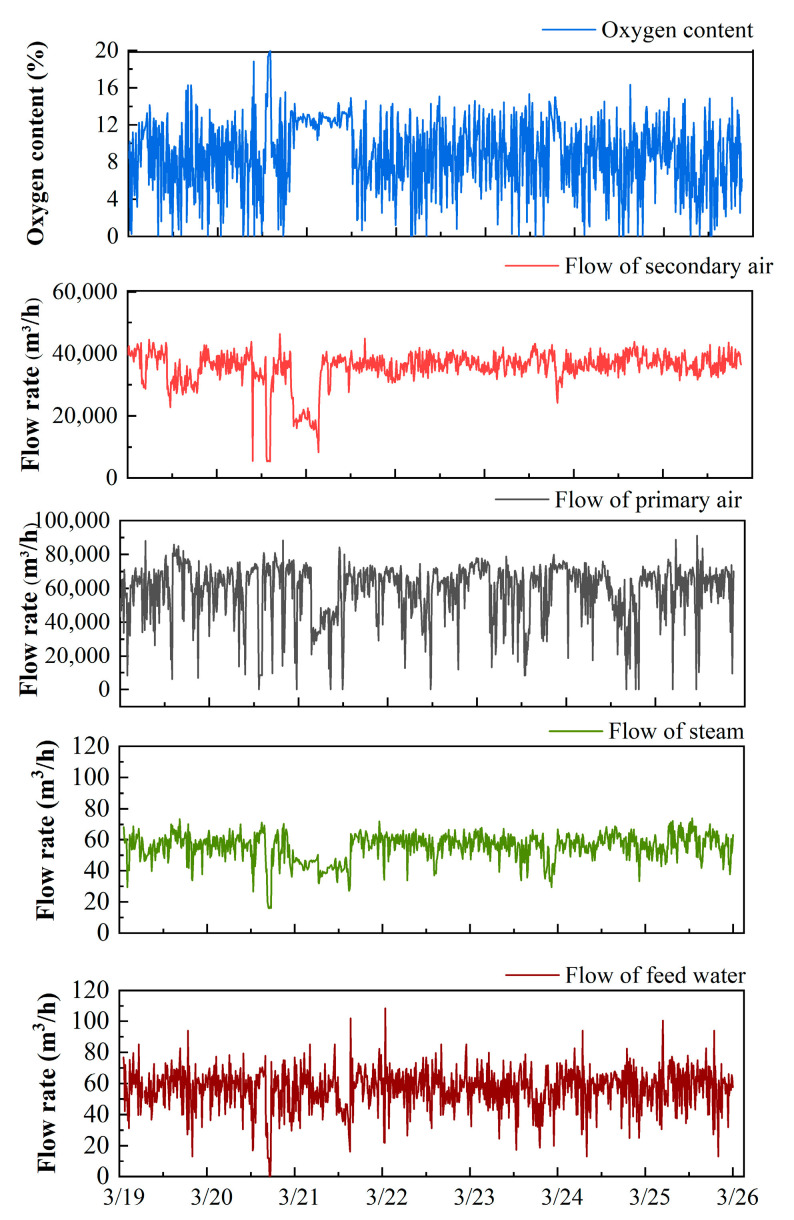
The trend for oxygen content, flow of primary air and secondary air, feed water, and steam.

**Figure 9:**
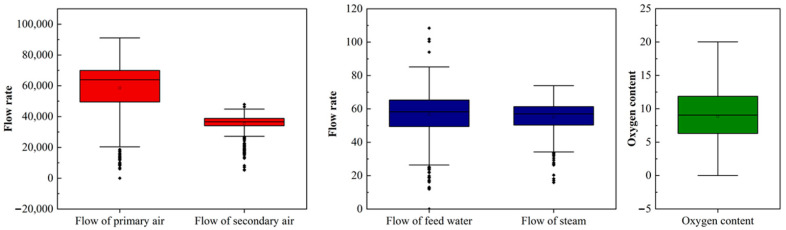
Comparison of the statistics for oxygen content, the flow of primary air and secondary air, feed water, and steam.

Figure 8 shows the trend for oxygen content, the flow of primary air and secondary air, feed water, and steam. Due to the long-term failure of the screw feeder, it is reasonable for the flow rates of the primary air and the feed water to decrease to zero. As shown in Figure 3 and Figure 8, a significant delay can be observed for the decrease of PCDD/F when the flow of the secondary air decreases. Additionally, the change of the flow of primary air lags behind the change of the flow of secondary air. The comparison of the statistics for the oxygen content, flow of primary air and secondary air, feed water, and steam are illustrated in Figure 9. The distribution of the flow of primary air is the same as that of secondary air. Additionally, the primary air flow is directly correlated with the mass transfer rate between waste and air. These results indicate that the flow of primary air is a better operating parameter for controlling PCDD/F emissions than the flow of secondary air. Regarding the PCDD/F emissions on 21 March, the oxygen content was maintained stably and above 10%, resulting in low PCDD/F emissions (≤0.1 ng I-TEQ/Nm^3^). However, at other times, the oxygen content fluctuated greatly from 0% to 20%, which corresponds to the great fluctuation of PCDD/F emissions and the flow of feed water and steam.

For specific daily analysis (Table S1, Supplementary Materials), it can be found that the PCDD/F emissions increase in the condition that the oxygen content fluctuate sharply or below 5% during the following times: 20 March (22:10–22:30); 21 March (3:30–6:30); 22 March (12:00–13:00, 15:30–16:20, 19:50–20:30); 23 March (21:30–22:00); 24 March (12:30–15:00); and 25 March (15:20–15:30, 16:10–17:00, 18:00–21:00). This is because low oxygen results in a high level of incomplete combustion soot, thereby promoting PCDD/F formation in de novo synthesis. On the other hand, PCDD/F emissions decreased to 0.1 I-TEQ/Nm^3^ under the condition that the oxygen content was stable and above 8%. Since high oxygen content promotes the formation of PCDD/F in the laboratory-scale incinerator, the influence of incomplete combustion products is larger than that of the oxygen content.

### 3.3. Correlation Analysis between PCDD/F Emissions and Operational Parameters

The correlation between PCDD/F emissions and operational parameters is analyzed by the Pearson coefficient and the maximal information coefficient in Table 2. There are no significant correlated variables with PCDD/F I-TEQ. All Pearson coefficients are less than 0.2. This result indicates that operational parameters have a weak linear correlation with PCDD/F emissions. As for the correlations analyzed by the maximal information coefficient, all variables have higher coefficients with PCDD/F emissions than the correlation analyzed by the Pearson coefficient. Moreover, the maximal information coefficients increase from 0.011 to 0.203, from −0.003 to 0.231 for bed pressure and pressure of secondary air, respectively. This result indicates that the relationship between operational parameters and PCDD/F emissions is more possible with a nonlinear relationship rather than a linear one. Correlation analysis indicates that there is no significant strong correlation (linear or nonlinear) between operating parameters and PCDD/F emissions. This is mainly because the formation of PCDD/F is directly related to many factors, such as temperature, oxygen content, pressure, and mass transfer rate. Only when there is a significant change in one of these factors will the PCDD/F emissions also change accordingly.

**Figure 10:**
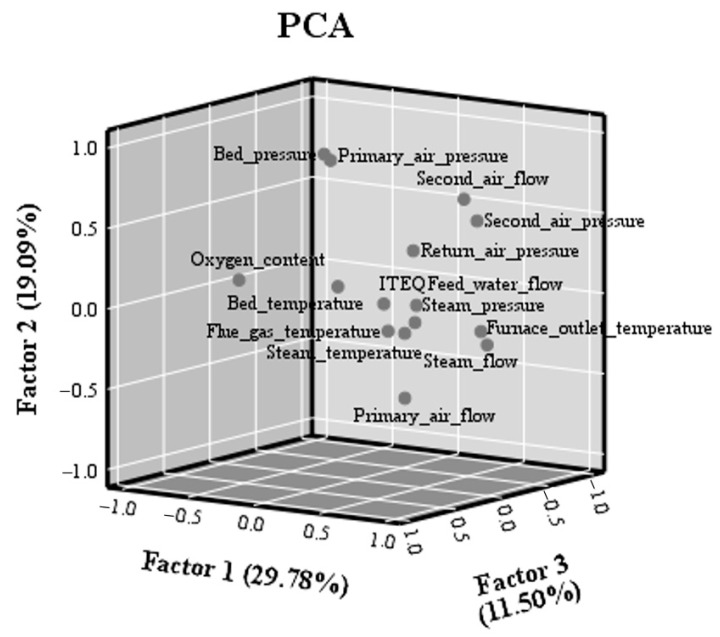
PCA analysis for 14 operating parameters and PCDD/F I-TEQ.

The relationship between 14 operating parameters and PCDD/F I-TEQ was investigated by PCA (Figure 10). The total contribution of the first three factors is only 60.36%, and the contribution of Factor 1 is 29.78%. This result is consistent with the 80% loading value of the first five factors in a previous study [31]. The results show that the first three factors do not represent most of the information of the sample, and the self-correlation between variables is small. In addition, the flow of steam has a high correlation with the temperature of the furnace outlet. A similar correlation between the pressure of secondary air and the flow of secondary air can be observed. Furthermore, the bed temperature, the furnace outlet temperature, steam pressure, and oxygen content were located with PCDD/F I-TEQ, indicating the close relationship. Moreover, the flow of secondary air, the flow of primary air, bed pressure, and pressure of secondary air were located far from PCDD/F I-TEQ. This outcome indicates the weak relationship between the above parameters and PCDD/F emissions.

To investigate the correlation between the temperature and PCDD/F emissions, the plummet of the operating parameters was introduced to describe the fluctuation. The plummet is defined as the temperature is 10% being below the mean in a short time. Generally, the plummet of the temperature of a furnace outlet is caused by the insufficiency of air or waste, resulting in incomplete combustion and a large number of organic pollutants. There are 43 minimum points for furnace outlet temperature illustrated in Figure 3. The plummet in the temperature of the furnace outlet signals the poor operation of the incinerator. Among the 43 minimums of the furnace outlet temperature, 29 values (67.4%) directly correspond to the maximum PCDD/F I-TEQ in Figure 3. This result indicates that the plummet is positively correlated to the increase in PCDD/F emissions. However, the other 14 plummets of the furnace outlet temperature do not match the maximum PCDD/F I-TEQ. This abnormal outcome may be the effect of APCD, which reduces PCDD/F emissions. In addition, the total count of the plummet of bed temperature is 13. Among them, 6 plummets (46%) correspond to the maximum of PCDD/F emissions. The other plummets do not correspond to the change in PCDD/F emissions. Therefore, compared with bed temperature, the furnace outlet temperature can better reflect the change of the incinerator operation.

### 3.4. Diagnosis and Feedback Control of PCDD/F

**Figure 11:**
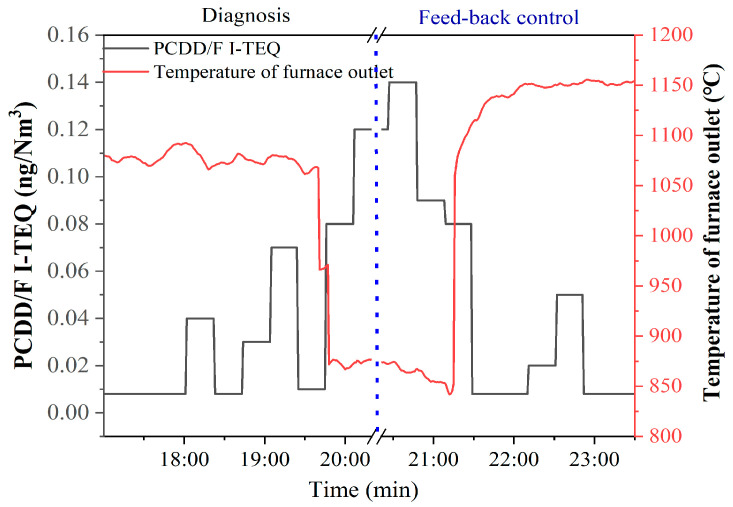
Diagnosis and feedback control of PCDD/F based on the furnace outlet temperature.

As shown in Figure 11, PCDD/F I-TEQ increases from 0.008 ng I-TEQ/Nm^3^ to 0.14 ng I-TEQ/Nm^3^ under the condition that the temperature of the furnace outlet decreases from 108 °C to 870 °C within 10 min. This result indicates that the plummet of the furnace outlet temperature can promote the formation of PCDD/F. The plummet of the furnace outlet temperature can be caused by the suspension of MSW feedstock, resulting in the incomplete combustion of the carbon matrix. A previous study reported that abundant PCDD/F was formed from the soot or carbon matrix [20], especially on the effect of metal catalysts (CuCl_2_, Fe_2_O_3_, etc.). Therefore, it is reasonable for the PCDD/F emissions to increase. Remarkably, the delay of the increase in PCDD/F emissions can be observed by comparing the increase of PCDD/F emissions and the decrease of the temperature. More specifically, when the temperature of the furnace outlet dropped to 870 °C, the PCDD/F emission simultaneously rose to 0.08 ng I-TEQ/Nm^3^ rather than the highest emission (0.14 ng I-TEQ/Nm^3^), which occurred later. This result indicates that the formation of PCDD/F is affected by the reaction time. Furthermore, the design of feedback control should consider the delay effect.

**Figure 12:**
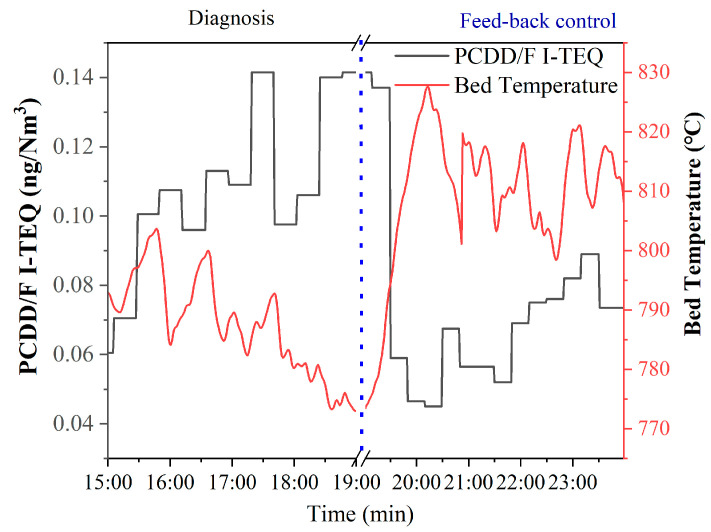
Diagnosis and feedback control of PCDD/F based on bed temperature.

**Figure 13:**
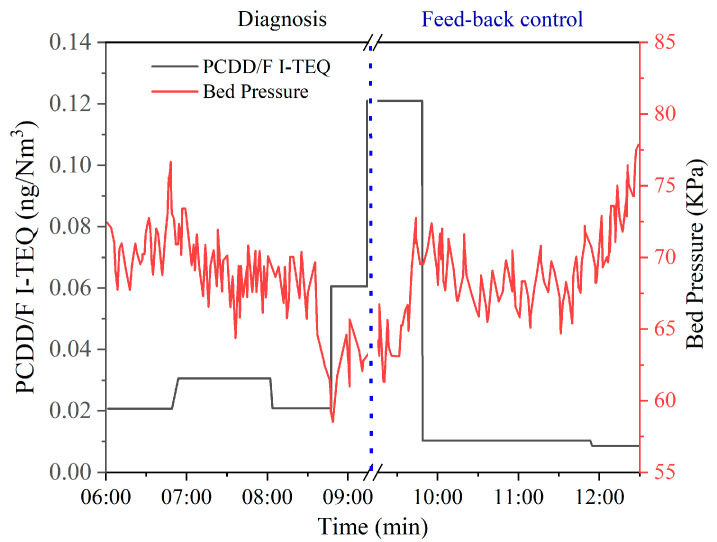
Diagnosis and feedback control of PCDD/F based on bed pressure.

As shown in Figure 12, PCDD/F emissions increase from 0.06 ng I-TEQ/Nm^3^ to 0.14 ng I-TEQ/Nm^3^ under the condition that the bed temperature decreases from 800 °C to 774 °C within 4 h. This result indicates that an increase in bed temperature over 800 °C can reduce the formation of PCDD/F. As shown in Figure 13, PCDD/F I-TEQ increases from 0.02 ng I-TEQ/Nm^3^ to 0.12 ng I-TEQ/Nm^3^ under the condition that the bed pressure decreases from 70 kPa to 60 kPa within 10 min. This result indicates that the plummet of bed pressure can promote the formation of PCDD/F. As for CFB, the low bed pressure results in a low air concentration gradient. Hence, the diffusion coefficient for the air to the waste is low, according to the Fick Law. The increase in PCDD/F emissions is reasonable for the decrease in the bed pressure.

Apart from the diagnosis of operational parameters, the feedback control of PCDD/F was constructed based on correlation analysis and equipment for the online monitoring of PCDD/F. As shown in Figure 11, the PCDD/F emissions decreased from 0.14 ng I-TEQ/Nm^3^ to 0.008 ng I-TEQ/Nm^3^ when the temperature of the furnace outlet returned to 1152 °C. Therefore, the equipment for the online monitoring of PCDD/F emissions can realize the feedback control of PCDD/F emissions. As a result, the reduction of PCDD/F emissions can be achieved. Thus, excessive emissions over the national standard can be prevented by adjusting the operating parameters. Moreover, due to the bed temperature outlet increasing from 774 °C to 826 °C within 1 h, PCDD/F emissions descended steeply from 0.14 ng I-TEQ/Nm^3^ to 0.05 ng I-TEQ/Nm^3^. When the bed pressure increased from 60 kPa to 77.5 kPa, PCDD/F emissions decreased from 0.12 ng I-TEQ/Nm^3^ to 0.008 ng I-TEQ/Nm^3^. These results indicate that adjusting the temperature and pressure parameters to the levels corresponding to PCDD/F emissions lower than 0.1 ng I-TEQ/Nm^3^ can be an effective method for controlling PCDD/F emissions under the national standard.

The above results show that the diagnosis and feedback control of PCDD/F emissions can be realized by the TD-GC-REMPI-TOFMS. As for industrial managers, the real-time PCDD/F emissions can be obtained through the online monitoring dioxin system. Thus, it is feasible to adjust operating parameters to reduce PCDD/F emissions in time. The optimization and guidance of incineration operating conditions have enabled the newly built CFB to meet the national standard of PCDD/F emissions.

## 4. Conclusions

To realize stable and effective online monitoring and feedback control of PCDD/F, the continuous 168 h measurement was carried out at the stack of a full-scale MSW incinerator. Then, the diagnosis and feedback control of PCDD/F was constructed on a newly built MSW incinerator. A drastic fluctuation of PCDD/F emissions could be observed during the long-term operation of MSWI, with 44% of PCDD/F I-TEQ below 0.1 ng/Nm^3^. Among the operating parameters, the furnace outlet temperature, bed pressure, and oxygen content are key operating parameters for controlling PCDD/F emissions. Maintaining the furnace outlet temperature to be higher than 800 °C, or the bed pressure higher than 13 kPa, or the oxygen content stably and above 10% are effective methods for reducing PCDD/F emissions. According to the analysis of Pearson coefficients and maximal information coefficients, there is no significant correlation between operating parameters and PCDD/F I-TEQ. Only when there is a significant change in one of these factors will the PCDD/F emissions also change accordingly. The feedback control of PCDD/F emissions was realized by adjusting the furnace outlet temperature, bed temperature, and bed pressure to control the PCDD/F to be less than 0.1 ng I-TEQ/Nm^3^. This study can provide effective and quantized guidance for controlling PCDD/F emissions under the national standard during the operation of MSWI. In the future, the control models between operating parameters and PCDD/F emissions will be established to achieve real-time automatic control of PCDD/F emissions to the national standard.

## Figures and Tables

**Table 1:** Statistics of operating parameters and PCDD/F concentrations.

Statistics	Min	Max	Mean	Standard Deviation
Bed temperature (°C)	724.56	921.42	835.59	29.02
Bed pressure (kPa)	−0.04	14.65	10.01	1.44
Flow of primary air (m^3^/h)	4186.58	82,527.50	58,456.06	14,329.15
Pressure of primary air (kPa)	0.00	15.40	11.31	1.37
Flow of secondary air (m^3^/h)	5316.99	43,428.07	35,527.49	5364.39
Pressure of secondary air (kPa)	−0.20	2.42	1.74	0.46
Pressure of return air (kPa)	0.00	19.21	17.86	1.39
Oxygen content (%)	0.32	19.75	8.82	2.97
Temperature of furnace outlet (°C)	274.21	894.21	780.59	66.89
Temperature of flue gas (°C)	158.44	270.78	193.05	15.20
Flow of feed water (m^3^/h)	0.00	84.53	56.64	9.86
Flow of steam (m^3^/h)	15.90	70.72	55.21	8.13
Temperature of steam (°C)	375.73	490.58	460.77	9.91
Pressure of steam (kPa)	−0.99	5.36	5.01	0.40
PCDD/F (ng I-TEQ/Nm^3^)	0.01	2.37	0.30	0.39

**Table 2:** Correlation between PCDD/F emissions and operational parameters.

**Variables**	**Bed Temperature**	**Bed Pressure**	**Flow of Primary Air**	**Pressure of Primary Air**	**Flow of Secondary Air**	**Pressure of Secondary Air**	**Pressure of Return Air**
Pearson Coefficient	−0.143	0.011	0.060	0.037	0.014	−0.003	0.100
Maximal Information Coefficient	0.166	0.203	0.160	0.203	0.184	0.231	0.127
**Variables**	**Oxygen Content**	**Temperature of Furnace Outlet**	**Temperature of Flue Gas**	**Flow of Feed Water**	**Flow of Steam**	**Temperature of Steam**	**Pressure of Steam**
Pearson Coefficient	−0.176	0.102	0.078	0.037	0.135	0.054	−0.054
Maximal Information Coefficient	0.183	0.182	0.231	0.158	0.181	0.171	0.134

## Data Availability

The authors declare that all data generated or analyzed during this study are included in the published article.

## Supplementary Materials

The following are available online, Figure S1: The analysis equipment for 1,2,4-TrCBz (TD-GC-TLI-TOFMS), Table S1: Original data of Oxygen content and Temperature of furnace outlet, and I-TEQ from March 19 to March 25.

## Sample Availability

Samples of the compounds are available from the authors.
