# Supplementary material for: Stable and Effective Online Monitoring and Feedback Control of PCDD/F during Municipal Waste Incineration
Source: Molecules. 2021 Jul 15;26(14):4290. doi: 10.3390/molecules26144290 (PMC8305602; doi:10.3390/molecules26144290)
Supplement: Supplementary file 1 [file molecules-26-04290-s001.zip › molecules-1242969-supplementary.pdf]

Stable and Effective Online Monitoring and Feedback Control of PCDD/F during Municipal Waste Incineration

Shijian Xiong [1], Fanjie Shang [2], Ken Chen [1], Shengyong Lu [1,*], Shaofu Tang [2], Xiaodong Li [1] and Kefa Cen [1]

[1] State Key Laboratory of Clean Energy Utilization, Zhejiang University, Hangzhou 310027, China; 11827072@zju.edu.cn (S.X.); 21927009@zju.edu.cn (K.C.); lixd@zju.edu.cn (X.L.); kfcen@zju.edu.cn (K.C.)
[2] Zhejiang Fuchunjiang Environmental Technology Research Co., Ltd., Hangzhou 311401, China; shangfanjie@163.com (F.S.); shaofutang263@163.com (S.T.)
* Correspondence: lushy@zju.edu.cn

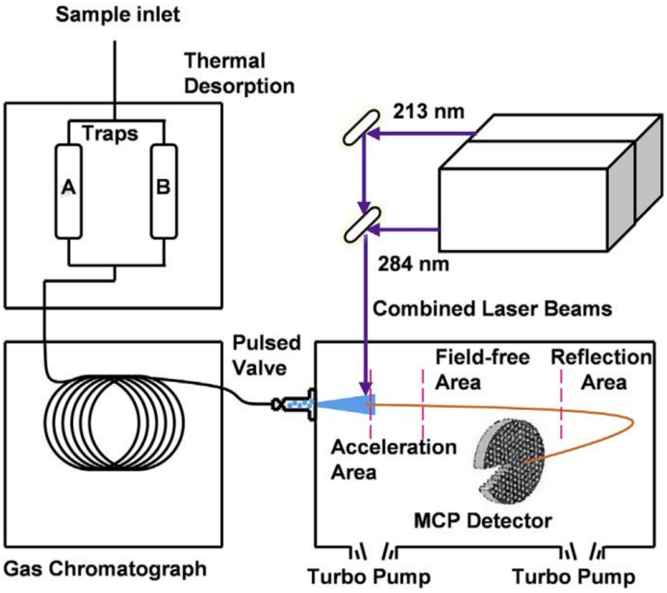

Figure S1. The analysis equipment of 1,2,4-TrCBz (TD-GC-TLI-TOFMS).

Table S1 Original data of Oxygen content and Temperature of furnace outlet, and I-TEQ from March 19 to March 25

Oxygen content	Temperature of furnace outlet	I-TEQ
4.81	829.71	0.36
5.99	791.82	0.70
4.53	825.03	0.06
14.26	729.01	0.03
13.01	667.61	0.06
7.96	770.85	1.02
9.52	812.91	0.36
4.83	809.35	0.80
4.30	815.72	0.97
10.07	783.39	0.48
5.16	780.30	0.28
9.76	845.96	0.55
4.69	833.52	0.28
8.03	840.48	0.31
10.93	740.41	0.06
8.28	801.43	0.04
11.75	811.08	0.16
10.73	758.67	0.01
11.44	791.94	0.06
11.83	772.08	0.08
12.93	767.65	0.09
9.95	759.10	0.06
9.83	785.93	0.01
9.75	744.46	0.26
9.21	742.78	0.38
8.18	827.36	0.48
9.40	725.73	0.68
11.87	741.97	0.68
10.03	781.46	0.48
2.72	773.52	0.28
11.16	803.91	0.36
10.26	812.93	0.53
6.96	747.52	0.41
11.83	725.87	0.26
5.28	830.80	0.80
6.30	786.87	0.75
10.54	755.72	0.21
8.97	783.61	0.23
9.41	814.76	0.36
3.55	850.72	1.68

8.31	831.41	2.37
2.52	851.91	0.97
9.33	842.76	1.29
7.68	818.12	1.48
7.45	804.25	0.87
3.39	854.32	0.60
4.42	894.21	0.85
9.95	792.07	1.17
6.21	812.25	0.77
8.58	820.43	0.85
7.21	825.31	0.14
7.56	834.90	1.58
8.61	719.62	1.09
11.09	799.12	0.99
5.70	841.95	0.36
7.40	820.27	0.04
15.56	561.38	1.26
6.46	767.89	1.06
5.36	753.79	1.02
4.93	807.09	1.97
11.05	780.72	1.06
9.42	764.38	1.36
8.86	791.15	1.46
6.28	848.22	0.68
11.61	688.43	0.23
4.50	838.18	0.23
8.35	817.15	0.70
7.60	816.79	0.80
6.58	815.36	0.60
5.00	779.78	0.50
5.59	849.13	0.65
11.24	748.00	0.77
10.50	735.09	0.46
6.13	811.98	0.38
9.79	782.68	0.33
10.54	801.43	0.23
6.62	821.26	0.16
9.79	772.48	0.31
7.67	730.79	1.04
8.42	820.10	0.55
4.49	815.02	0.55
11.91	734.85	0.06
3.51	826.22	0.43
9.40	735.87	0.48

11.01	802.38	0.31
7.82	785.83	0.28
9.54	812.12	0.38
9.20	805.36	1.34
8.54	788.56	0.43
9.32	834.86	0.14
10.93	780.23	0.04
11.79	719.49	0.63
9.36	823.97	0.46
6.38	817.48	0.31
8.30	832.65	0.19
10.58	818.30	0.77
5.19	822.29	0.41
11.33	747.97	0.68
0.32	744.36	0.90
5.63	789.53	0.77
10.77	795.65	1.26
8.94	778.70	0.70
3.86	802.80	0.11
7.60	808.93	0.90
9.40	764.63	0.83
11.64	708.85	0.36
11.12	664.91	0.90
4.33	821.90	0.47
11.64	722.07	0.23
5.13	864.93	0.69
5.52	817.14	0.75
7.27	830.67	0.42
5.00	860.10	0.25
2.25	877.22	0.23
5.48	858.86	0.23
8.07	864.52	0.07
11.90	795.48	0.10
14.45	633.08	0.05
19.40	399.50	0.10
19.75	299.68	0.05
17.00	274.21	0.28
8.55	762.02	0.12
10.10	752.97	0.90
8.58	743.91	0.59
8.27	836.85	0.71
8.46	838.64	0.95
4.54	860.82	0.46
10.66	769.26	0.43

8.06	670.00	0.68
12.10	763.04	0.84
3.59	815.10	1.00
5.71	859.73	0.95
9.48	757.43	0.68
6.11	858.64	0.07
7.33	850.87	0.10
8.49	756.25	0.04
11.97	764.23	0.06
13.52	667.01	0.04
12.30	660.85	0.06
11.87	783.21	0.06
12.73	704.44	0.06
11.98	731.64	0.06
12.61	726.73	0.08
12.61	721.46	0.09
12.77	714.49	0.11
12.69	709.34	0.09
12.73	707.12	0.09
12.89	685.73	0.09
12.61	693.02	0.11
12.10	745.99	0.09
13.09	697.35	0.09
13.01	687.46	0.11
12.30	705.50	0.11
12.22	746.11	0.12
12.14	736.73	0.09
12.61	730.34	0.09
11.94	733.45	0.09
11.94	723.36	0.12
11.24	734.47	0.11
12.07	758.51	0.11
13.09	573.46	0.12
12.26	641.79	0.12
13.09	658.24	0.11
12.85	668.73	0.11
12.77	677.62	0.14
12.81	676.09	0.12
12.61	681.20	0.12
12.58	698.09	0.12
12.42	708.55	0.11
11.75	721.22	0.11
12.37	718.65	0.12
12.63	706.44	0.11

12.50	704.74	0.06
12.18	721.99	0.04
12.77	726.27	0.03
14.22	587.79	0.03
11.79	726.09	0.06
12.61	722.29	0.06
13.32	680.08	0.08
13.36	665.24	0.06
13.24	671.07	0.03
13.17	672.83	0.06
12.02	738.38	0.06
13.59	675.47	0.04
13.13	619.47	0.04
14.06	505.45	0.04
9.08	700.59	0.06
9.40	768.50	0.04
7.32	798.31	0.06
8.78	808.29	0.06
1.82	822.98	0.06
7.13	811.10	0.03
9.12	818.22	0.04
8.50	818.52	0.04
3.82	855.88	0.06
10.70	789.12	0.03
5.55	799.48	0.04
9.87	736.97	0.03
5.87	839.19	0.04
6.81	868.34	0.06
7.56	810.79	0.06
6.54	775.61	0.08
8.68	775.14	0.12
9.91	779.14	0.14
9.94	811.57	0.04
5.99	810.15	0.01
6.30	819.35	0.11
7.24	845.52	0.09
9.04	797.37	0.09
10.34	760.83	0.04
5.40	847.76	0.03
7.29	813.67	0.01
8.22	812.34	0.03
9.00	692.10	0.06
9.76	732.34	0.09
9.05	815.36	0.24

5.63	823.34	0.04
10.58	742.99	0.33
10.54	777.60	0.12
4.93	832.17	0.06
6.30	830.69	0.02
2.61	806.93	0.04
9.09	788.35	0.03
9.87	774.31	0.03
9.05	814.22	0.06
8.34	829.30	0.19
7.74	820.10	0.22
6.62	820.62	0.03
7.52	828.17	0.04
10.56	827.47	0.01
9.12	761.35	0.02
8.57	817.33	0.03
12.02	817.15	0.04
6.68	720.08	0.01
1.18	847.72	0.05
10.18	759.30	0.03
7.00	814.96	0.07
4.61	818.41	0.21
7.67	791.92	0.15
9.87	829.37	0.01
5.43	828.77	0.15
8.54	801.69	0.05
4.57	798.72	0.03
9.79	797.77	0.07
9.20	800.04	0.03
5.44	818.85	0.03
5.66	817.08	0.03
7.25	821.49	0.15
8.14	776.79	0.30
10.69	777.77	0.03
6.38	724.88	0.66
8.38	833.77	0.24
11.90	739.44	0.03
5.72	775.82	0.48
11.71	776.37	0.50
13.24	625.60	0.01
14.02	647.41	0.72
7.55	726.36	0.09
5.52	805.75	0.09
11.67	791.12	0.15

7.47	816.02	0.05
9.71	784.19	0.01
5.64	778.94	0.13
8.66	825.47	0.09
8.81	799.83	0.09
8.61	807.82	0.05
8.35	809.90	0.03
7.75	798.54	0.07
11.60	776.78	0.05
6.46	768.56	0.03
4.69	786.05	0.07
9.64	781.87	0.05
8.30	851.11	0.01
10.42	799.29	0.05
6.46	816.07	0.03
8.73	820.62	0.01
12.34	794.92	0.01
12.34	773.13	0.03
10.88	718.82	0.01
7.60	848.87	0.07
12.06	771.40	0.03
9.05	802.48	0.09
9.01	780.69	0.07
7.03	797.71	0.03
10.78	836.82	0.01
10.20	736.22	0.02
10.84	775.15	0.26
12.18	753.07	0.17
12.57	816.19	0.04
7.32	832.65	0.03
5.24	856.13	0.03
10.93	787.16	0.02
11.17	780.10	0.07
8.86	825.98	0.03
6.81	865.19	0.03
10.70	815.90	0.03
12.77	762.83	0.09
5.59	828.72	0.09
7.13	830.96	0.11
11.01	747.77	0.01
10.19	805.76	0.09
11.36	728.09	0.04
8.10	817.02	0.04
4.41	812.55	0.02

7.21	820.82	0.15
11.95	790.94	0.28
6.62	812.01	0.35
10.61	796.64	0.02
11.75	686.56	0.08
5.95	796.64	0.08
8.80	812.79	0.04
7.52	804.03	0.07
7.29	845.00	0.04
7.72	808.03	0.04
10.78	794.63	0.12
5.90	796.07	0.56
10.21	775.08	0.61
7.98	808.44	0.27
9.20	820.84	0.37
6.34	801.40	0.50
2.88	804.03	0.87
7.95	812.97	0.22
11.91	671.28	0.84
9.36	766.74	0.14
11.24	801.88	0.15
11.32	779.48	0.40
13.79	611.21	0.24
6.49	757.69	0.06
7.67	766.27	0.20
13.00	725.81	0.07
8.02	834.42	0.45
13.79	670.29	0.07
6.54	727.02	0.09
7.21	779.43	0.01
4.18	831.36	0.20
10.18	818.38	0.51
9.71	866.80	0.28
7.72	806.25	0.17
8.85	828.97	0.20
7.70	834.66	0.06
6.21	760.65	0.28
9.44	776.42	0.14
6.11	797.82	0.04
11.79	738.34	0.38
5.05	855.41	0.40
14.46	664.48	0.32
13.63	621.19	0.01
12.65	678.89	0.04

11.59	732.83	0.06
12.65	707.04	0.03
14.41	584.68	0.04
13.36	618.89	0.02
11.86	721.79	0.01
11.77	678.61	0.03
11.20	702.58	0.01
6.89	814.62	0.02
5.95	836.46	0.01
8.53	835.17	0.04
8.30	776.16	0.12
6.97	812.55	0.04
8.82	790.76	0.10
6.50	778.06	0.25
9.64	800.11	0.22
8.77	794.08	0.15
6.15	875.35	0.10
6.57	861.67	0.15
9.13	775.46	0.19
6.88	766.83	0.33
8.18	813.82	0.55
9.01	772.95	0.14
10.73	803.37	0.50
6.54	830.71	0.17
4.49	843.32	0.07
2.02	834.02	0.10
8.42	779.40	0.12
10.42	739.91	0.02
9.64	831.57	0.79
1.35	829.78	0.53
9.94	776.72	0.20
10.78	792.84	0.40
10.43	799.54	0.19
9.08	783.02	0.17
5.38	796.59	0.15
9.21	820.77	0.04
12.15	775.63	0.03
10.68	773.74	0.03
8.11	796.80	0.01
10.25	754.73	0.03
12.53	775.83	0.03
9.60	779.34	0.01
7.52	807.51	0.04
3.43	844.66	0.06

8.15	802.91	0.04
7.24	790.85	0.01
9.44	807.84	0.04
7.37	832.58	0.04
8.07	857.03	0.01
7.47	816.30	0.09
7.95	810.50	0.09
10.48	791.39	0.06
6.35	806.20	0.04
2.88	847.50	0.06
9.52	849.57	0.09
6.42	839.44	0.08
2.01	855.10	0.01
5.70	826.16	0.30
4.50	826.37	0.59
7.60	833.59	0.37
8.51	817.84	0.27
5.20	836.29	0.27
11.75	804.15	0.50
6.19	817.83	0.22
8.07	831.78	0.24
11.79	796.46	0.24
9.64	734.68	0.03
8.69	773.51	0.06
5.39	825.66	0.09
8.42	850.42	0.07
5.60	806.40	0.09
8.69	813.31	0.30
3.92	820.91	0.14
10.15	770.38	0.64
7.13	792.72	0.51
13.64	674.83	0.24
11.67	763.08	0.27
10.15	805.24	0.12
9.29	850.85	0.12
8.82	820.53	0.01
10.34	793.32	0.08
8.27	838.72	0.08
7.63	755.69	0.10
11.44	724.01	0.01
8.94	803.26	0.06
10.58	763.71	0.07
11.29	741.39	0.04
7.72	823.06	0.03

8.78	851.36	0.01
11.87	790.70	0.03
9.64	723.18	0.04
8.12	796.53	0.03
10.07	793.49	0.04
12.07	740.18	0.06
8.85	804.83	0.09
9.16	814.21	0.19
9.71	780.40	0.07
10.43	743.13	0.01
9.40	749.52	0.10
4.97	823.72	0.17
7.87	865.79	0.19
8.42	840.17	0.09
9.48	823.87	0.20
6.92	826.05	0.12
4.97	852.82	0.10
8.66	807.42	0.07
13.99	717.55	0.07
6.58	838.85	0.32
5.91	833.76	0.12
9.64	827.62	0.04
4.46	832.17	0.24
3.94	845.74	0.15
7.48	841.46	0.51
10.42	797.17	0.04
7.16	775.69	0.07
6.70	790.93	0.03
2.02	880.76	0.40
3.71	843.03	0.38
3.12	843.46	0.30
6.93	863.69	0.20
3.79	859.75	0.53
5.68	840.17	0.14
9.72	802.06	0.40
11.87	771.92	0.61
6.61	786.98	0.58
8.86	795.36	0.32
13.48	699.89	0.90
5.70	754.93	0.51
4.04	864.58	0.66
10.42	737.85	0.22
1.47	825.10	0.69
5.91	819.15	0.77

3.12	830.09	0.77
8.11	852.00	0.82
10.03	749.72	1.87
9.05	812.10	1.77
7.86	829.32	1.77
7.67	816.20	1.39
5.20	815.38	0.63
2.89	857.98	1.41
7.76	788.54	0.92
11.60	764.93	1.04
11.56	709.42	0.58
8.14	812.21	2.31
8.93	742.11	1.54
8.46	761.89	1.18
9.17	805.27	0.86
6.97	832.26	1.59
5.99	825.39	0.79
11.25	735.52	1.35
11.75	692.29	1.30
6.26	796.37	1.12
5.52	837.12	0.71
